# Bioinspired Postural Controllers for a Locked-Ankle Exoskeleton Targeting Complete SCI Users

**DOI:** 10.3389/frobt.2020.553828

**Published:** 2020-11-16

**Authors:** Jemina Fasola, Romain Baud, Tristan Vouga, Auke Ijspeert, Mohamed Bouri

**Affiliations:** ^1^Laboratory of Cognitive Neuroscience (LNCO), School of Life Sciences, Ecole Polytechnique Fédérale de Lausanne (EPFL), Geneva, Switzerland; ^2^Biorobotics Laboratory (BIOROB), School of Engineering, Ecole Polytechnique Fédérale de Lausanne (EPFL), Lausanne, Switzerland; ^3^Translationnal Neural Engineering (TNE), School of Engineering, Ecole Polytechnique Fédérale de Lausanne (EPFL), Geneva, Switzerland

**Keywords:** balance, posture, controller, exoskeleton, position-control, standing, paraplegic

## Abstract

Several lower-limb exoskeletons enable overcoming obstacles that would impair daily activities of wheelchair users, such as going upstairs. Still, as most of the currently commercialized exoskeletons require the use of crutches, they prevent the user from interacting efficiently with the environment. In a previous study, a bio-inspired controller was developed to allow dynamic standing balance for such exoskeletons. It was however only tested on the device without any user. This work describes and evaluates a new controller that extends this previous one with an online model compensation, and the contribution of the hip joint against strong perturbations. In addition, both controllers are tested with the exoskeleton TWIICE One, worn by a complete spinal cord injury pilot. Their performances are compared by the mean of three tasks: standing quietly, resisting external perturbations, and lifting barbells of increasing weight. The new controller exhibits a similar performance for quiet standing, longer recovery time for dynamic perturbations but better ability to sustain prolonged perturbations, and higher weightlifting capability.

## Introduction

Lower-limb exoskeletons have gained much interest in the last decade. This growing interest is mainly driven by the aim of enhancing human performance and improving neuromotor rehabilitation. Therefore, developing novel features to improve user safety, mobility and autonomy is a constant research challenge. In the field of wearable robotic systems for complete spinal cord injured (SCI) patients, walking is the main function targeted by the majority of lower-limb exoskeletons. Balance management while walking and standing is generally performed by the user with the help of crutches, and thus impairing the use of their hands for other activities. Very few full-mobilization exoskeletons are able to self-stabilize (Donati et al., 2016; Gurriet et al., 2018; Rex Bionics, 2020), and thus, allow to free the user's hands. This comes at the cost of low walking speed and an important overall weight. In addition, none of them can climb stairs for example. Standing and walking balance are essential functions to promote exoskeleton usage during daily activities, that however should not come to the detriment of other features. In daily-life activities, manual tasks and environmental interactions happen mainly while standing (e.g., shaking hands, grabbing an object, drinking) rather than walking. Therefore, a valuable trade-off would be to enable the usage of the hands during standing for exoskeletons actuated only in the sagittal plane. While the fore-aft balance could be actively regulated, the lateral stability can be maintained passively if the space between the feet is large enough, thanks to the wider base of support (BoS).

Humans are constantly adjusting their posture to act against gravity and are capable to resist to moderate internally generated or environmental perturbations using body coordination only (i.e., without stepping). To counteract these perturbations, proactive and reactive forms of postural movements are generated by the sensorimotor system to keep the center of mass (CoM) within the BoS (Rogers and Mille, 2018). Thus, coping with unexpected and self-generated perturbations requires a robust postural controller. As of today, there is no full-mobilization exoskeleton, position-controlled, actuated only in the sagittal plane capable of maintaining a standing posture with users that do not have any control of their lower limbs. Therefore, our goal is to develop a postural controller for TWIICE One, a lower-limb exoskeleton for complete SCI users.

Several research groups work on partial assistance during stance in the goal to improve the balance of people with incomplete SCI. Most of these control strategies are using torque control (Rajasekaran et al., 2015; Emmens et al., 2018; Farkhatdinov et al., 2019; van Asseldonk et al., 2019). These studies mimic the most common postural strategies highlighted by Winter (1995): the ankle, the hip, and their combined strategies. However, the limited number of degrees of freedom of TWIICE One, especially its locked ankles, does not allow to directly adopt these control strategies. For that reason, a bioinspired approach was adopted to identify and then implement the elicited postural strategies on TWIICE One. From this approach, two postural controllers have been developed. This case study aims to present and characterize the performance of these two postural controllers enabling a complete SCI user to stand without crutches.

These controllers are potentially useful for the current generation of full-mobilization exoskeletons, because they do not need torque control in the joints, or load cells in the feet. The hardware can then be kept minimal, so the device can be simpler, less expensive and more robust.

## Bioinspired Approach: Learn From a Passive Exoskeleton

**Figure 1:**
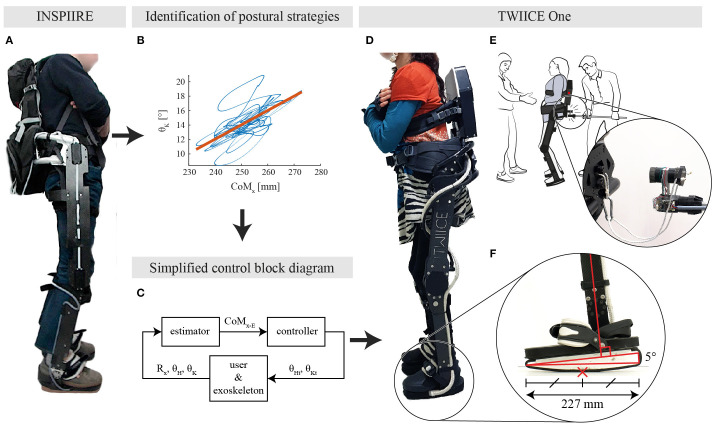
**(A)** Healthy participant standing while being constrained by INSPIIRE, a passive exoskeleton. **(B)** Identified relation between the knee angle and the CoM_x_ for a typical young healthy participant in the eyes closed condition from Fasola et al. (2019). **(C)** Overview of the controller block diagram. CoM_x−E_ is the estimated projection of the center of mass, R_x_ represents the foot angle with respect to the ground line, while θ_H_ and θ_K_ are the hip and knee angles, respectively. **(D)** TWIICE running with the knee controller (BKC) and a complete SCI user. **(E)** Overview of the experimental setup, with the experimenter in the back, and the spotter in front. The experimenter interacts with the exoskeleton through the instrumented stick. **(F)** Close-up view on the TWIICE foot, with the rounded sole and the 5° wedge. The red cross is the position of the CoP, at the middle of the foot in this case. This is considered as the “horizontal” position of the foot.

In a previous study, we observed how young healthy participants adapted their postural control strategies when wearing a passive exoskeleton (Fasola et al., 2019). This device, called INSPIIRE (see Figure 1A), has the same kinematic constraints as TWIICE One, and fully curved foot soles, see Figure 1F. It has been found that healthy adults mainly manage their postural balance by flexing and extending their knees to move the contact point along the anteroposterior axis while standing quietly inside a passive locked-ankle exoskeleton (Figure 1B). Based on segmental analysis, this strategy is referred to as a vertical strategy, meaning that the trunk and the shank orientations move in phase and thus the whole body moves along the vertical axis (Nashner and McCollum, 1985). In case of more consequent perturbations, the hip strategy was used to maintain balance. During the hip strategy, the shank rotation is not sufficient to keep the CoM over the base of support; therefore, the trunk rotates in the opposite direction to compensate and reposition the CoM.

Drawing inspiration from this human sensorimotor adaptation, a novel postural position controller has been implemented and tested on TWIICE with no user (Baud et al., 2019). This controller regulated the balance with a proportional-derivative (PD) controller, acting on the angle of the knee, and fed with the estimated CoM position (Figure 1C). This “knee controller” was able to manage the balance of TWIICE autonomously and resist to short perturbations.

In this article, the controller developed by Baud in 2019 is tested with a complete SCI user. In addition, an extended version of this knee controller is described and compared to the baseline knee controller. It is designed to resist stronger long-term perturbations.

## Postural Control Framework

### Lower Limb Exoskeleton “TWIICE ONE”

The two postural controllers have been implemented on the lower limb exoskeleton TWIICE One 2018, Figure 1D. This exoskeleton is similar to the version of 2016 introduced in (Vouga et al., 2017). The mechanical design and the control framework are the same, while the actuators are more compact and more powerful (Billet et al., 2019). TWIICE One provides two active DoFs per leg for the flexion/extension of the hip and knee joints in the sagittal plane. The ankle joints are locked at 90°. To match the experimental conditions of the passive exoskeleton (Fasola et al., 2019), the foot soles profile has been modified. It is then fully curved (no flat part in the middle) to prevent passive postural stability (Figure 1E). Its 0.65 m radius is the same as the previous study, which is smaller than the height of the CoM of the test-pilot, so passive equilibrium is not possible. The sole is 227 mm long, which corresponds to a maximum range of movement of 242 mm for the contact point, when the sole is rolling on the floor. The top part of the exoskeleton foot is tilted forward by 5°, so when the middle of the foot is in contact with the floor, the shank axis has a 5° angle with respect to the vertical axis (Baud et al., 2019). The soles are covered with a rubber layer, to prevent slippage when standing.

The width of the BoS is 244 mm, measured between the two outer faces of the sole skates.

The elevation angle on the sagittal plane (also called “pitch angle”) of the exoskeleton's foot is estimated from the inertial measurement unit (IMU) data with a simple complementary filter algorithm similar to Gui et al. (2015). Since the IMU is aligned with the exoskeleton, only a single gyroscope axis, and two accelerometer axes are required. The elevation angle of the thigh and trunk segments are computed from the estimated foot elevation angle and the joints encoders angles. Instead of using the trunk IMU as in Baud et al. (2019), the IMU located in the left foot is used instead. It is expected to increase the performance for two reasons. First, when swinging fore-aft the whole body, the foot is the location with the lowest linear acceleration, which makes the state estimation more accurate. Second, there is more vibration in the trunk, that is less rigid and in a cantilever configuration, which can generate closed-loop self-sustained uncontrolled oscillations.

The embedded computer of TWIICE collects at 1 kHz the data from the inertial measurement unit (IMU) and the joints encoders.

TWIICE is not a certified commercial device, but it is deemed safe, so it is very unlikely that the pilot could be harmed in this experiment. Its hardware and operation are fully documented, and a failure mode and effects analysis (FMEA) was performed. It was also inspected and approved by the organizers of the CYBATHLON (Riener, 2016), as a prerequisite to participate in this event.

### Proposed Postural Controllers

#### Baseline Knee Controller

**Figure 2:**
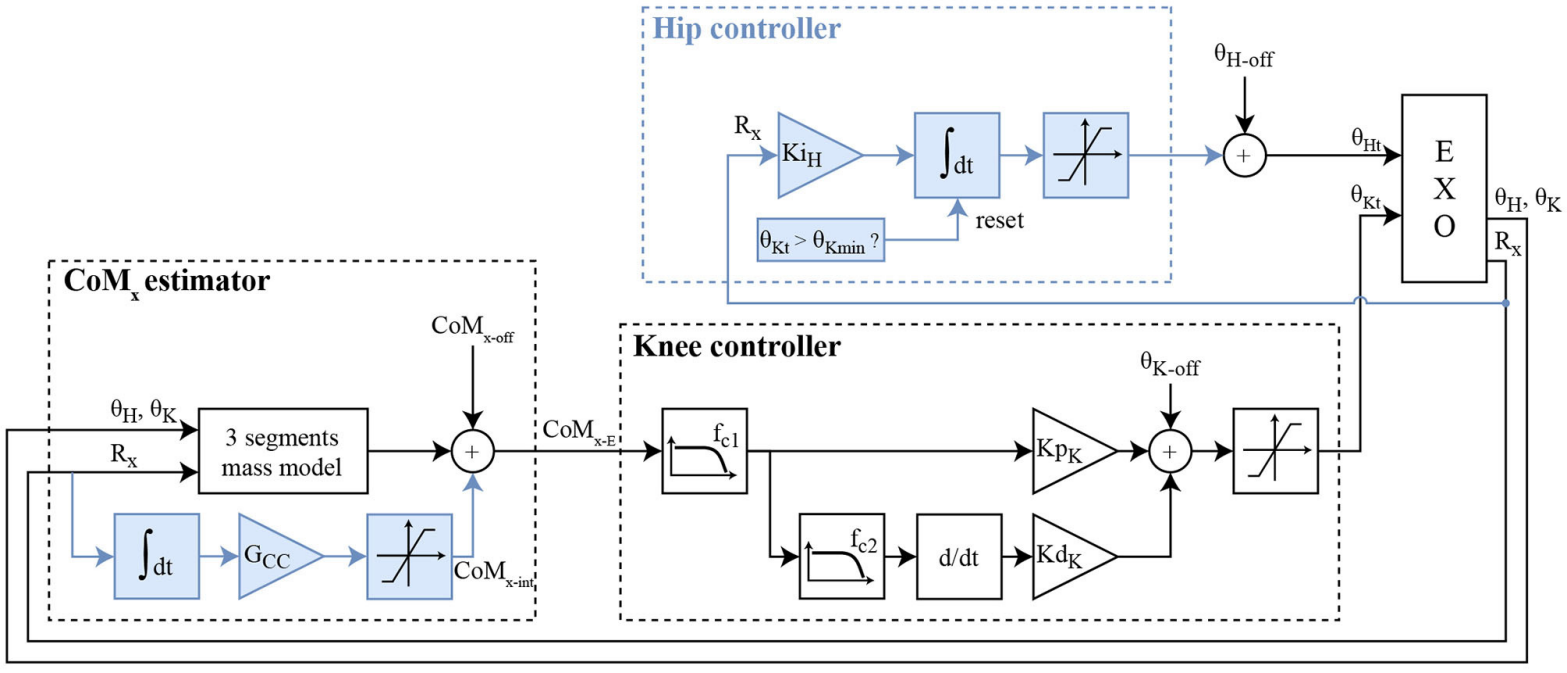
Block diagram of the two controllers. BKC is made of the CoM_x_ estimator and the knee controller (white blocks). EKC is made of the improved CoM_x_ estimator, the knee controller, and the hip controller (all the blocks). The blue boxes are thus the additions of EKC. CoM_x−E_ corresponds to CoM_x−E1_ for BKC, and CoM_x−E2_ for EKC. CoM_x−off_ represents the offset added to the estimated projection of the CoM in the sagittal axis. θ_K−off_ and θ_H−off_ are, respectively, the baseline offsets of the knee and the hip angles.

The “Baseline Knee Controller” (BKC) regulates the balance with a proportional-derivative (PD) controller, setting the angle of the knee, and fed with the CoM position (Figure 2). This “Baseline Knee Controller” was described, simulated and experimentally tested (Baud et al., 2019). The knees are flexed proportionally to the estimated position of the CoM. This makes the foot sole rotate forward and backward, and move the point of contact with the floor. Since the sole is only in contact with the ground at one point, this point corresponds also to the center of pressure, x_CoP_, on the anteroposterior axis. CoM_x_ is the position of the projection on the ground of the CoM, on the anteroposterior axis. Its origin is defined at the middle of the foot when it is in contact with the ground. In this BKC controller, the CoM_x_ estimation is computed using a simple 2D model consisting of 3 segments (foot to knee, knee to hip, trunk including the head). The trunk length was measured on the user, while the shank and thigh lengths were obtained from the 3D model of the exoskeleton. The masses were obtained by summing the pilot's and exoskeleton's segments. The masses of the user segments were estimated from the full bodyweight using the mass repartition from Fang et al. (2017), considering the data corresponding to “chronic SCI ≥ 3 years” and “BMI < 25.” Finally, an offset, CoM_x−off_, is added to the estimation of CoM_x_ to obtain CoM_x−E_, which is called CoM_x−E1_ in the BKC case. This offset is necessary because the model is not accurate.

CoM_x−E_ is first filtered by a low-pass filter with a cut-off frequency fc_1_, then fed into a proportional-derivative controller (PD) with the parameters Kp_K_ (proportional part gain) and Kd_K_ (derivative part gain). Before differentiation, the signal is filtered by a stronger low-pass filter with a cut-off frequency fc_2_. This gives a knee flexion angle, which is offset by θ_K−off_ to increase the flexion, and thus avoids hyperextension of the knee when the output of the BKC controller is negative. For safety, the value is finally clamped to the range [2 to 40°]. The hip joint is fixed at the angle θ_H−off_.

A pilot study with the BKC controller has demonstrated its ability to make a complete SCI user stand dynamically with TWIICE. However, it was performing poorly for the task of grabbing heavy objects (several kilograms), unless they were close to the body. The first reason is that the CoM_x−E1_ computation is not accurate since it does not consider the added mass. The other reason is that the controller is managing the balance by moving the position of the CoP along the foot length, but this does not work in the case the added weight shifts the CoM beyond the span of the feet.

#### Extended Knee Controller

The extended knee controller (EKC) is based on the BKC, but with two additions to overcome the two aforementioned issues (Figure 2, blue boxes). The first change is the extension of the CoM_x_ estimator, to adapt the model online when a constant perturbation (added mass or horizontal force) arises. This is done with a gain (G_CC_) and an integrator of the R_x_ value, R_x_ being the foot angle with respect to the ground line (or the elevation angle of the foot, minus 90°). The output of this integrator is added as a variable offset to the CoM_x−E_ calculation, called CoM_x−E2_ for this controller (Figure 2, CoM_x_, blue boxes). The idea is that in case of a permanent perturbation, the CoP will move durably, closer to an end of the foot, which decreases the robustness against perturbations in this direction. Continuously increasing the CoM_x−E_ offset will increase the correction of the PD controller, until the sole starts to roll in the other direction. This means that if a steady-state exists, the center part of the foot will be in contact with the floor. Adding an integrator to the regulator to make a PID controller instead would not have the same results. This will not be proved analytically here, but intuitively, in case of constant perturbation with BKC and a PID, the steady-state will be reached when the CoM_x−E1_ reaches zero, but the CoP will probably not be in the middle of the foot, so the robustness would be lower in one direction.

The second change is the addition of the hip contribution when the knee reached the full extension. In case the knee reaches the full extension, an integrator with a gain Ki_H_ will gradually increase the hip flexion angle, to bring the trunk forward, and thus shift the CoM toward the front (Figure 2, Hip controller, blue boxes). This flexion angle is limited to 60° for safety. If the knee angle is not saturating, the integrator value is reset to zero smoothly at a 2°/s rate. This conservative low value was selected to make sure it does not interfere the with the knee control and avoid oscillations.

Simulations have been performed with the same Simulink simulation environment described in Baud et al. (2019). The goal of this model is to check the proper operation of the controller, i.e., keeping the body standing without falling. The stability is assessed from the values of CoM_x−E_ and the foot elevation angle, which should remain close to 90°. This model contains a three weighted segments model, lumping together the user body and the exoskeleton, the feet rolling on the floor with no slippage. It is subject to a horizontal perturbation force, applied at the hip joint axis, with a square profile: 0 N, then 20 N forward for 8 s, then 0 N again. The parameters have been set as follows: Kp_K_= 340°/m, Kd_K_= 170°/(m/s), no filtering. For BKC, Ki_K_= 340°/(m.s) and G_cc_= 0 m/(°.s). Ki_K_ is the integral coefficient if the knee PD controller is replaced by a PID. It is not required and will not be used on the actual device, but it allows a clearer comparison between the results obtained with BKC and EKC, thanks to the eventual cancellation of the CoM_x−E_ steady-state error (it will show that the effect of the integral component of EKC is not equivalent as using a PID with BKC). For EKC, Ki_K_= 0°/(m.s) and G_cc_= 0.000002 m/(°.s).

**Figure 3:**
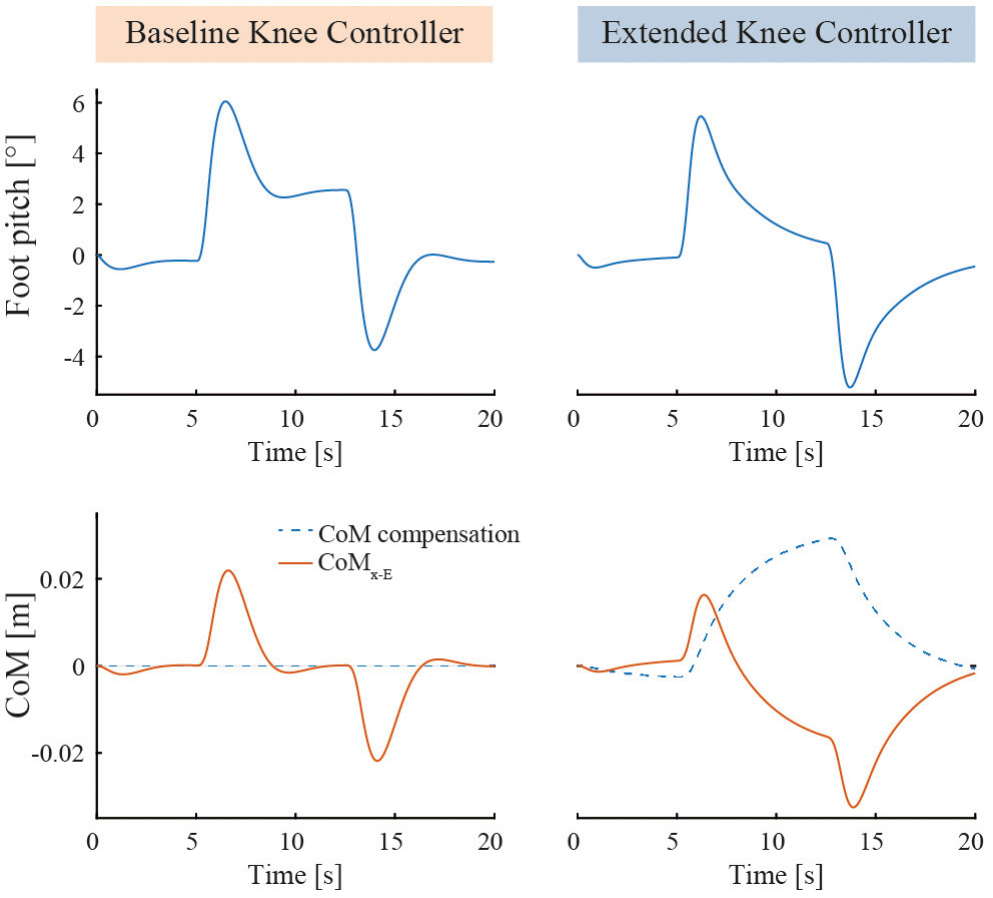
Simulation results comparing BKC (with PID) and EKC when subject to a constant horizontal perturbation force. The horizontal pushing perturbation starts at *t* = 5 s, stops at *t* = 13 s, has an intensity of 20 N and a ramping time of 0.5 s.

The results are exposed in Figure 3. It can be noticed that both BKC and EKC both maintained the standing balance despite the perturbation. Both the foot elevation angle and CoM_x−E_ exhibit minimal oscillations. With BKC, the system is stable during the perturbation, but the foot is not horizontal at steady-state (2.6°), which leaves less control leeway for further pushes. In fact, the CoP reaches the end of the foot when |R_x_|>10°. With EKC, R_x_ also reaches approximately 6° when the perturbation is applied but then returns slowly to 0° (CoP at the middle of the foot), which results in having the same room for maneuver in both directions again. While returning to the horizontal position, R_x_ follows an exponential function with a time constant of 2.4 s (*R*^2^ = 0.997). This means that a stronger long-term perturbation should rise slowly, not quicker than a few seconds, otherwise the integral action of EKC will not compensate fast enough to avoid the fall.

**Figure 4:**
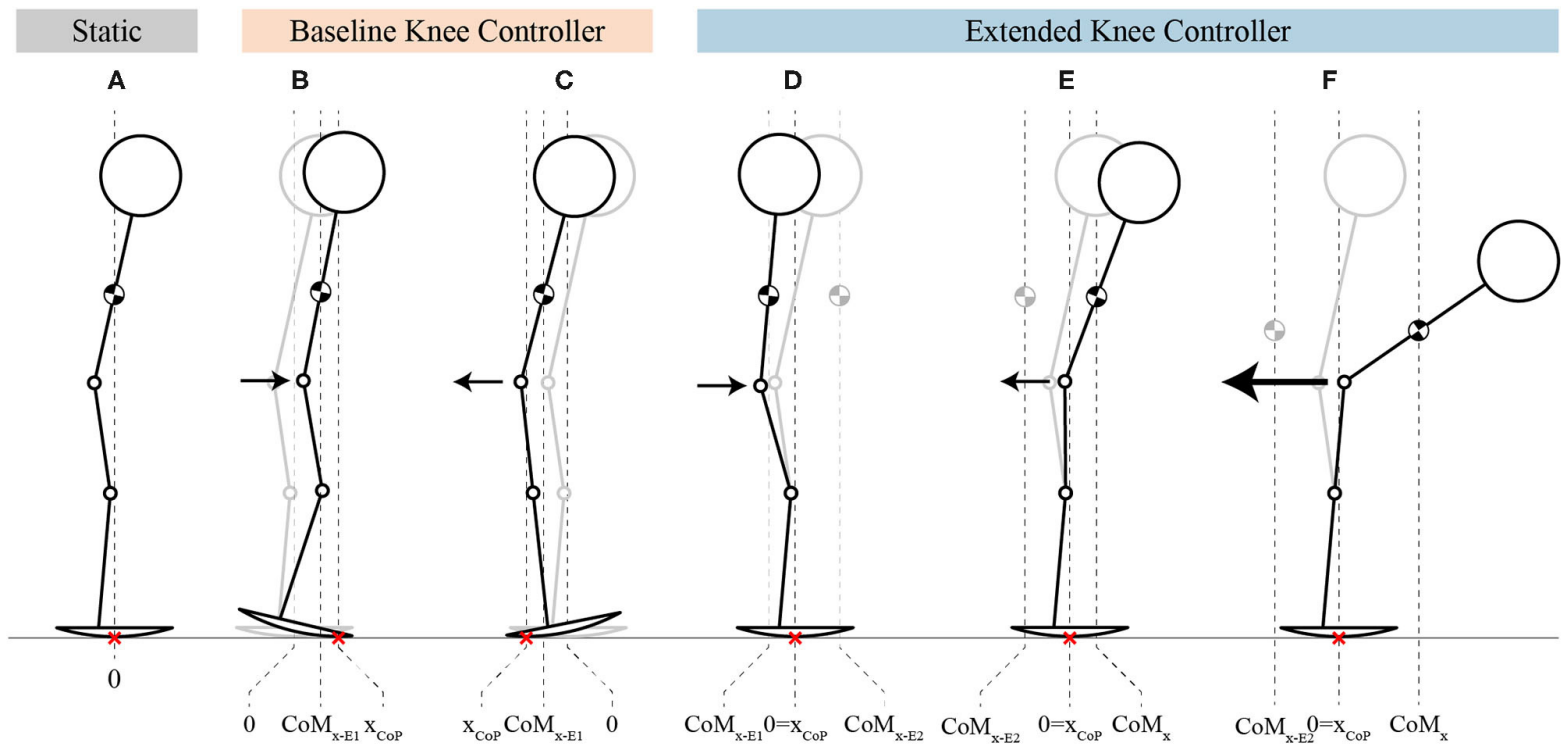
Stick-figures showing the behavior of both controllers, at steady-state. **(A)** The stick-figure represents the initial rest position. **(B,C)** The stick-figures show the CoP displacement (x_CoP_) and the behavior of the knees during push and pull perturbations, respectively, for BKC. **(D–F)** The stick-figures represent the behavior of EKC by showing how the position of the CoP is kept at the center of the foot thanks to the torso compensation for moderate push and pull perturbations, as well for strong pull perturbation. The arrows indicate the direction of the perturbation while their size is proportional to the perturbation amplitude. The red crosses represent the position of the point of contact with the ground, which is equivalent to the CoP in the sagittal axis (x_CoP_). The gray stick figures in background are the initial equilibrium position, same as in **(A)**.

The general behavior of the two controllers at steady-state can be seen in Figure 4. In the BKC case, the system statically resists the perturbation by keeping the CoP more in the front (Figure 4B) or in the rear of the foot (Figure 4C). In the EKC case, the system resists statically by keeping the CoM toward the back (Figure 4D) or toward the front (Figure 4E), such that the CoP is at the middle of the foot. In the last case (Figure 4F), the pulling force is stronger, and the knee reached the full extension and cannot extend more. The hip then flexes to move the CoM even more in front.

## Methods

### Test-Pilot

Both postural controllers were tested with one chronic (10 years post-injury) and functionally complete SCI participants (ASIA A) with a lesion at the T10 level. She is 158 cm tall and weighs 45 kg. She has no contraindications for the use of an exoskeleton (strong spasms, contractures, low bone density, or cognitive deficiency) and uses regularly a passive verticalization device. She will be called “test-pilot” of the lower limb exoskeleton. She had a previous experience with the standing balance with two preliminary sessions, the exoskeleton running a provisional controller, similar to the current implementation of BKC. She gave informed consent to participate in the test sessions.

Only one participant was involved because this experiment aims assessing the performance of the device, not the user. The guidelines on assistive devices by the Swiss Ethics Committees on research involving humans (Swissethics, 2018) specify that exploratory works (evaluating “beta prototypes,” their overall operation, the function or robustness of the sensors and actuators, etc.) are not subject to the swiss law about research on humans.

### Protocol

The tuning of the controller parameters occurred during a dedicated session, 10 days before the actual experiment.

The experiment was composed of four tasks: quiet standing, pulse and static perturbations, and object lifting perturbation. The test-pilot was instructed to keep her arms crossed and look straight at a cross on the wall in front of her during the whole experiment. The floor is made of hard linoleum floor, with virtually no rolling resistance.

At all times, there were one spotter in front and one behind the pilot to catch her in case of loss of balance, since the controllers will not trigger a step when the stability margin is exceeded. The spotters' hands were very close to the exoskeleton handles or the pilot's body to ensure quick grabbing in case of loss of balance. Contact only occurs in case of loss of balance. The usual harness, cable and support frame could not be used because the cable would disturb the balance, probably positively and biasing the results.

#### Tuning Session

The regulator gains were first set to zero to disable the closed-loop control. θ_K−off_ was fixed arbitrarily, then CoM_x−off_, θ_H−off_ and were obtained by hand-tuning such that CoM_x−E1_ is zero when the exoskeleton stands still in the unstable equilibrium position, while the middle of the sole in contact with the ground. This procedure was repeated several times, to maximize θ_K−off_ under the condition that the posture is comfortable for the test-pilot.

Then, the low-pass filters and the PD parameters were tuned to maximize the disturbance rejection performance while no self-sustained oscillations or vibrations can be observed. Finally, Ki_H_ and G_CC_ were tuned to the highest value that does not generate self-sustained oscillations.

#### Quiet Standing

For both controllers, 1 min of quiet standing was performed in order to compare the sway amplitude without any perturbation.

#### Pulse Perturbations

The goal of the second task of the experiment consists in evaluating the responsiveness and stability of both controllers, when the exoskeleton is subject to short and high-intensity horizontal perturbations. The back part of the exoskeleton was pushed and pulled horizontally at the pelvic height (960 mm above the ground level) with a stick operated by an experimenter behind the pilot, so that she cannot expect the pulses (Figure 1E).

As in Emmens et al. (2018), the perturbation amplitude is defined by the push/pull force multiplied by the perturbation duration. The experimenter keeps the duration of the pulses short and as constant as possible. As long as the user is swinging, the LED on the backpack is red, and the experimenter will not interfere with the movement. When the sway velocity of the trunk (computed by time-derivation of the trunk elevation angle, obtained with the foot IMU and the joints encoders) remains below 0.015 rad/s (0.86°/s) for more than 2 s, the LED on the backpack turns green again, and a new perturbation can be applied. The perturbations are applied randomly by the experimenter. The supervision laptop counts the perturbations and sorts them into the weak/medium/strong categories for both directions, to help the experimenter applying all types of perturbations.

The stick is instrumented with a load cell, mounted with a stiff string such that it can push (posterior perturbation) and pull (anterior perturbation) the exoskeleton, or apply virtually no force when the pusher is not in contact and the string is loose. A custom amplifier and sampling board is also mounted on the stick, based on the ADS1146 chip (Texas Instruments, United States). It is wired to the exoskeleton embedded computer with four loose thin wires (0.129 mm^2^ copper section) to apply only minimal parasitic force on the exoskeleton. This allows the exoskeleton to log the load cell signal with the same time base as the exoskeleton data, to avoid the manual synchronization step after the experiment.

#### Static Pull and Push Forces

To assess the performance of both controllers during prolonged perturbations, the maximum horizontal force that is sustainable before losing balance was measured in both directions. The experimenter pushes with the instrumented stick, increasing slowly, and monotonically the force, until static equilibrium is lost. This procedure was repeated 3 times, and then was reiterated also 3 times by pulling the test-pilot backward. The user is caught and brought back to the vertical position by the experimenter at the end of each trial, so the recovery cannot be evaluated.

#### Object Lifting Perturbation

Finally, to define the anterior static margin of stability in a situation close to an actual use case, the test-pilot was asked to lift a barbell in front of her, and raise it gently at the shoulder height with the arms straight forward, then lower it down. Raising starts with the barbell at the lowest possible height, in contact with the legs. The mass of the barbell was changed from 0 kg (i.e., weight of the arms only) to 6 kg with increments of 2 kg. Each mass was lifted once. The task was failed if the spotters had to catch the test-pilot to prevent the fall, or if the test-pilot is unable to complete the task in <1 min. Unlike the previous test, the recovery back to the vertical position with no load is part of the task. The pilot then has no assistance at all from the experimenters while lifting, hovering and lowering. As opposed to the previous tasks, the participant has to use her arms for a simulated activity. This is the main reason why a manikin could not replace an actual participant in this protocol.

### Data Analysis

The analysis of the stability is performed using the CoM_x−E1_ metric, because there was no extra instrumentation that could measure the actual CoM_x_, and CoM_x−E2_ would be irrelevant when considering the static pull and push perturbations.

For quiet standing, a high-pass filter was applied to CoM_x−E1_ to discard potential position shift due to the slow head movement of the test-pilot. Then, the root mean square (RMS) of the CoM_x−E1_ was used to evaluate the amplitude of body sway for both controllers. For the pull and push task, perturbations with a duration deviating more than 0.1 s from the median duration were excluded. Thus, for each controller, only responses with similar perturbation duration were analyzed. Then, the pull and push perturbations were sorted each in three categories based on the distribution of the perturbation magnitude. These categories were the same for the two controllers.

The main assessment metrics were the recovery time and the maximal perturbation magnitude that the controllers can handle in both directions. The recovery time was defined as the time needed after a perturbation for the CoM_x−E1_ velocity to fall below a threshold set to 0.005 m/s. This threshold value was selected because it was the highest that still considered the oscillations due to the perturbations of the first category. A moving average filter with a span of 10% of the total number of data points was applied to the CoM_x−E1_ derivative. The maximal sustainable pulse perturbation amplitude in both directions was defined by the maximum perturbation amplitude that does not result in a loss of balance.

For the maximum sustainable pull and push force, the average of the 3 peak forces in each direction was computed.

## Results

The results of the parameters tuning session are shown in Table 1.

**Table 1:** Controller parameters values.

**Parameter**	**BKC-value**		**EKC-value**
CoM_x−off_		0.04 m	
f_c1_		20 Hz	
f_c2_		5 Hz	
θ_K−off_		8°	
θ_H−off_		0°	
Kp_K_		420°/m	
Kd_K_		110°/(m/s)	
Ki_H_	0°/(°.s)		0.3°/(°.s)
G_cc_	0 m/(°.s)		0.000002 m/(°.s)

### Quiet Standing

The oscillation frequency is similar in both cases: 0.60 Hz for BKC and 0.63 Hz for EKC. It was computed by finding the frequency of the highest peak in the Fourier transform of the CoM_x−E1_ signal. The RMS of the body sway is also similar (0.31 mm for BKC and 0.38 mm for EKC).

### Pulse Perturbations

**Figure 5:**
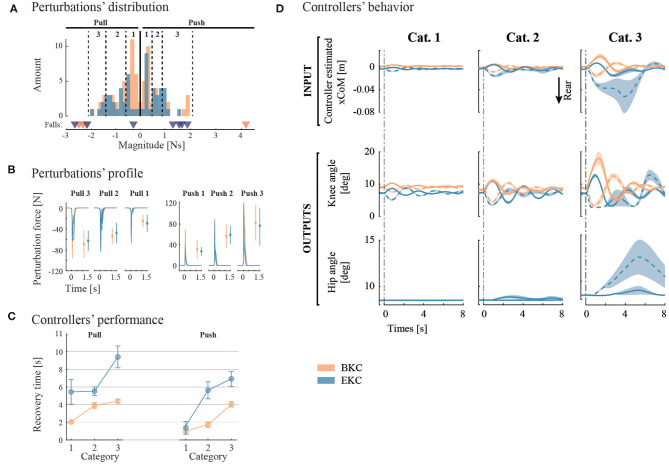
Pulse perturbation results and controllers' outputs. **(A)** The perturbations of similar magnitude are grouped in 3 categories for each direction. The thresholds of each category are represented by the vertical dotted lines. Underneath the histograms, perturbations that led to a fall are presented by a triangle. **(B)** Individual perturbation force profiles are shown by category. In addition, the average and standard deviation of the maximum perturbation forces are shown with error bars. **(C)** Controllers' performance is evaluated with the recovery time. **(D)** average system response of the two controllers subject to the 3 categories of perturbation Magnitudes. Solid lines represent posterior perturbations, while dotted lines denote anterior ones. Overall, the colors correspond to the controllers (orange for BKC and blue for EKC).

For BKC, 74 perturbations were applied, resulting in 4 fall initiations and 1 exclusion. For EKC, the test-pilot underwent 63 perturbations, including 7 fall initiations and 1 exclusion. The distribution of the perturbations' magnitudes and perturbations' forces by category are shown in Figures 5A,B. The average perturbation duration was 0.18 ± 0.006 s and 0.19 ± 0.006 s for BKC and EKC, respectively.

To characterize the robustness of the controllers, we determined the maximum anterior (pull) and posterior (push) perturbation amplitude the controllers can bear before a fall starts. For BKC, the maximal anterior perturbation magnitude that can be sustained is about 2 N.s. Beyond that threshold, 3 backward falls were recorded (see Figure 5A, orange triangles). The threshold for posterior perturbations is between 2 and 4 N.s, as a push with a magnitude of 4 N.s triggered a frontal fall. For EKC, the maximal anterior perturbation magnitude is also around 2 N.s. Two perturbations above this threshold triggered a backward fall. The maximal threshold for posterior perturbations is between 1.2 and 1.6 N.s. Indeed, 4 falls were observed when the perturbation magnitude was above this threshold (see Figure 5A, blue triangles). It is important to note that the falls were in the backward direction although the perturbations were posterior (pushes). In summary, BKC is more robust than EKC for posterior perturbations, while they perform similarly for anterior perturbations.

To assess the performance of the controllers, the average recovery time has been extracted and plotted on Figure 5C. BKC recovered faster in all conditions [mean 2.95 s, 95% CIs (2.55, 3.34)] than EKC [mean 5.49 s, 95% CIs (4.65, 6.34)].

The average system response is shown on Figure 5D. For the first and second perturbation categories, the response is similar, although the oscillations last longer with EKC. There are more differences for the third category. The pulling perturbations for EKC are producing a larger deviation of the CoM (~4 cm instead of ~ 2 cm for the other conditions), because the full extension of the knee was reached, and lowered the control capability. This is only the case for EKC, because initially, the knee was less flexed (the steady-state was not exactly the same), so there is less margin before the full extension is reached. It is also noticeable that even for the pushing perturbation, the hip contribution is used. This is because the oscillations have a high amplitude and low damping, this is why the system also reaches the backward position and result in saturating the knee angle in full extension and starts using the hip contribution.

### Maximum Static Push and Pull Forces

The maximum pushing force that can be sustained is higher for EKC [mean 75.07 N, 95% CIs (66.25, 83.90)] than for BKC [mean 13.69 N, 95% CIs (5.29, 22.09)]. The maximum pulling force is also higher for EKC [mean 27.92 N, 95% CIs (11.86, 43.97)] than for BKC [mean 13.26 N, 95% CIs (10.39, 16.13)]. EKC can endure higher static forces when the test-pilot is pushed forward than pulled backward (unpaired *t*-test *p* < 0.001), while there is no effect of perturbation direction for BKC (unpaired *t*-test *p* = 0.84).

### Object Lifting Perturbations

**Figure 6:**
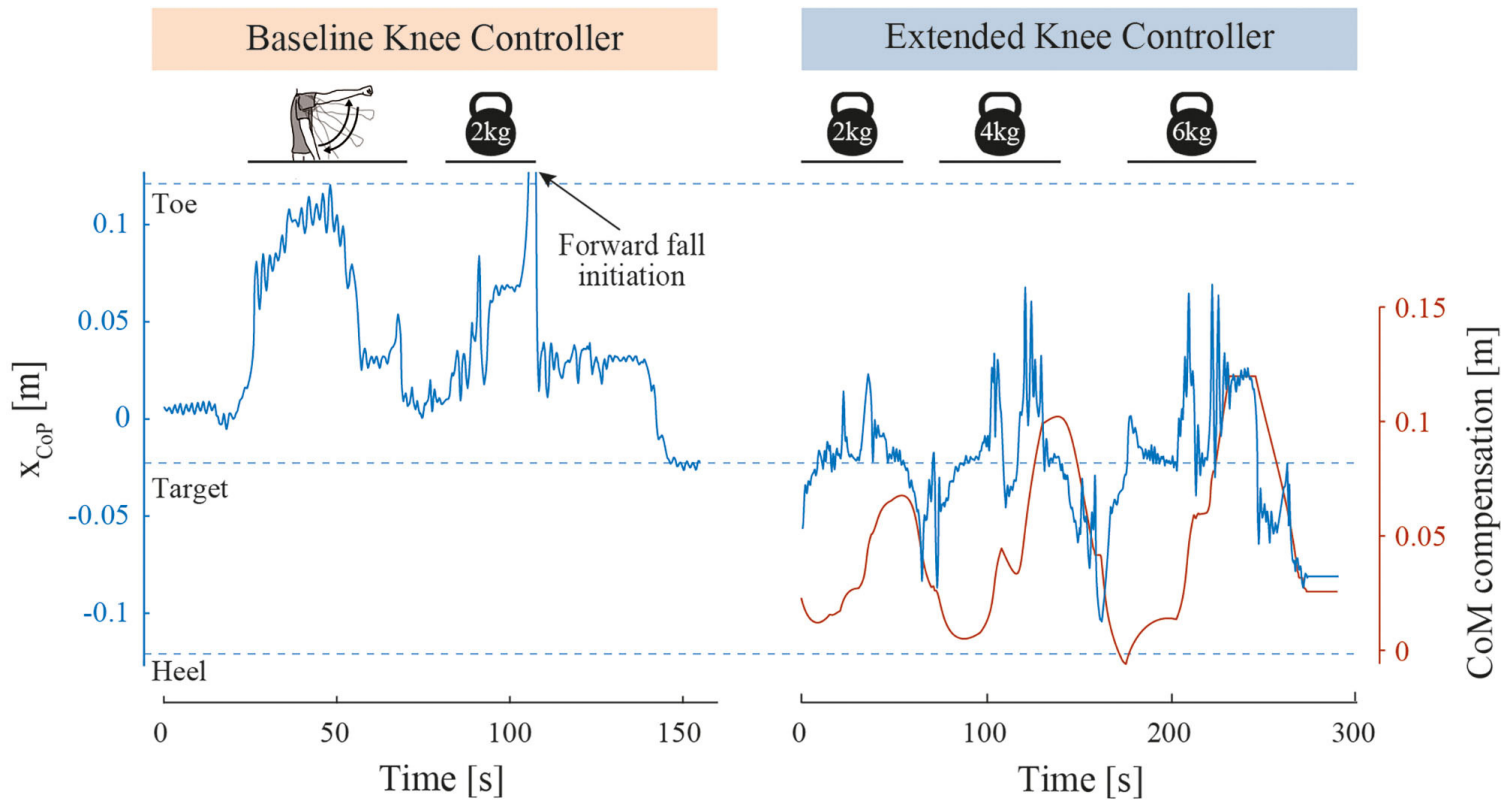
Object lifting perturbation results. Foot contact point position (x_CoP_) along the curved soles during object lifting perturbations. For BKC, the x_CoP_ of one successful arm lift and one failed barbell lift is shown. When the toe tip has been reached, the pilot fell forward, was caught by the spotters and therefore the x_CoP_ quickly returned close to zero. The barbell was then removed, and the system stabilization is highlighted by the x_CoP_ returning to the target position. For EKC, the x_CoP_ of 3 successful barbell lifts with increasing weights is represented, as well as the CoM compensation.

The results of this test are visible in Figure 6. With BKC, the test-pilot could lift her arms but failed to lift the 2 kg barbells because she started falling forward before reaching the shoulder height, even though the ascent was slow. With EKC, the test-pilot could successfully lift the 2, 4, and 6 kg barbells. The time for the pilot to perform each movement (lifting and lowering) is shown in Table 2.

**Table 2:** Time required to perform raising and lowering, for the barbell test.

**Condition**	**Raising time [s]**	**Lowering time [s]**
BKC, 0 kg	18	19
BKC, 2 kg	4*	N/A
EKC, 2 kg	15	17
EKC, 4 kg	17	21
EKC, 6 kg	16	21

* *The starred value denotes failure (fall initiation) before completion*.

## Discussion

The goal of this study was to test and compare two postural controllers with a low-actuator count exoskeleton and a complete SCI pilot. Both controllers were able to manage quiet standing with almost no body sway and to cope with anterior-posterior perturbations. In that respect, postural adaptation strategies observed in healthy participants with a passive exoskeleton have been successfully transferred onto an active full-mobilization exoskeleton. This results in the first exoskeleton with only two degrees of freedom per leg able to balance during standing with a complete SCI user. Overall, the EKC controller was more performant, although the recovery time is slightly slower.

For pulse perturbations, EKC damps the oscillations more slowly due to its integrative behavior, and thus has higher recovery times. Moreover, the falling direction was not always the same as the perturbation direction. Since the knees can flex more than they can extend in the actual configuration, it would possible to increase the posterior margin of stability by increasing the knee offset angle (θ_K−off_). However, this would imply that the hip flexion angle should also be increased to remain balanced, which results in an unnatural crouch standing. This also causes more load on the interfaces and in particular the trunk belt, which was reported to be an uncomfortable posture by the test-pilot during the tuning session. Overall, since the arms do not help to support the trunk through the crutches, the upper belt of the exoskeleton maintaining the torso should be sufficient and comfortable.

For static perturbations, EKC sustained higher pushing forces thanks to the torso adjustment and repositioning of the CoM, while there was no significant difference when pulling. It is important to note that EKC could resist even higher pulling forces, just by increasing the value of the maximum flexion of the hip, and if these forces change slowly. This would however be even more difficult to recover from.

**Figure 7:**
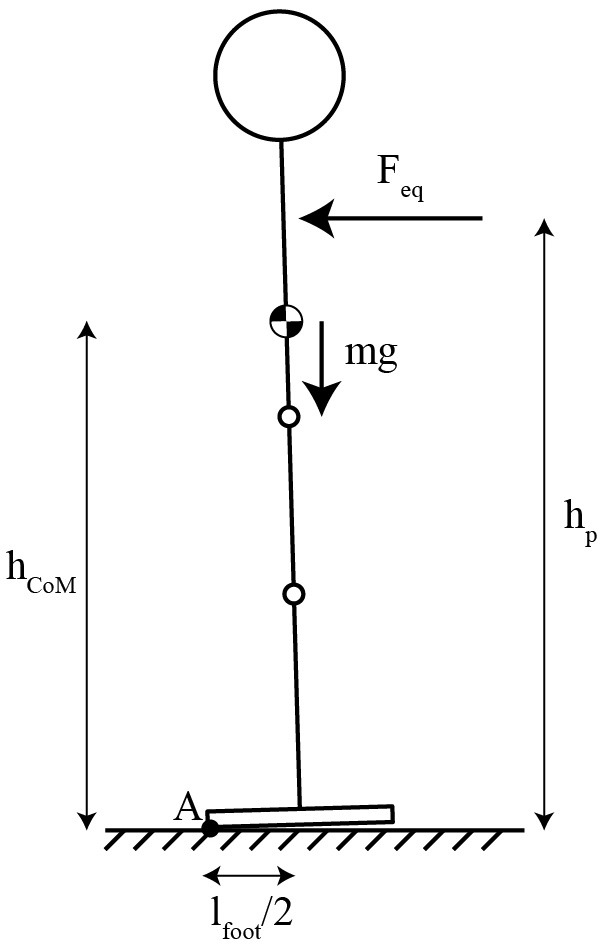
Equivalent system for the static balance calculation with flat feet.

The main motivation for the curved sole is to walk by rolling the foot on the floor, to compensate for the lack of a mobile ankle joint. However, it also enables the use of these balance controllers, which could give more stability than passive balance with a flat sole of the same length. In practice, this ability was limited by the clamping on the knee and hip angles. The theoretical maximum force that the system could resist without falling in the same conditions with flat feet can be computed by the simple static equilibrium model depicted in Figure 7. At equilibrium:

$$\begin{array}{l} \sum \tau_{A}\;=\;0\\ \Rightarrow \frac{l_{foot}}{2}mg\;=\;F_{eq}h_{p}\\ \Leftrightarrow F_{eq}\;=\;\frac{\frac{l_{foot}}{2}mg}{h_{p}}\;=\;70.2\;N \end{array}$$

where, τ_A_ is the torque at the pivot point A, l_foot_ is the length of the foot, m is the mass, g is the gravity, F_eq_ is the equivalent force applied and h_p_ is the height of the perturbation application. So, the EKC can resist a higher pushing force (75.07 N) than the passive balance with a flat foot (70.2 N, see equation), but this is not the case with the pulling force (27.91 N). This limitation comes from the fact the knee cannot overextend, and that the hip joint was limited to 60° of flexion.

The static perturbations assessment gives us some functional insights on how much the pilot, while standing in the exoskeleton, could pull and push an object during daily activities such as opening a door, reaching for a pack of water on a supermarket shelf or closing a car trunk. The current EKC controllers could make this kind of activities possible without the need for crutches.

Another functional assessment was the object lifting task. With the EKC controller, the user is able to manipulate a heavy object far from his body, but again, this is only possible if the movements are slow, otherwise the point of contact with the ground may reach one end of the foot, and the user will start falling. Nevertheless, the EKC controller would for example enable to drink from a 1L bottle without worrying about balance management. To facilitate the user to apprehend the ability of the exoskeleton to manage balance, acoustic or haptic feedback could be given when the CoP is close to the limits, so the user could, for example, decelerate the movement. Sensory feedback, in addition to being warning signals could also promote embodiment, and thus facilitate acceptation of the device (Pazzaglia and Molinari, 2016; Beckerle et al., 2018). More extensive training with the device and the controller is necessary to further improve the performance and to apprehend the behavior the exoskeleton should follow in case of risk of fall. The main limitations of this study are that these controllers have been tested with only one test-pilot and in a controlled environment. It would be interesting to observe how the performance varies as function of the ground texture and inclination, as well as with different users.

The main limitation of EKC is that the compensator adapts slowly to a permanent disturbance. Increasing the gain G_cc_ is not possible since it leads to an oscillatory behavior. An alternative would be to reuse the three segments model and associate it to a Kalman filter. This would allow to estimate the CoM_x_ offset quicker without inducing increasingly large oscillations.

For an actual use with the ADLs, the safety is of the utmost importance. The major concern is that there is no stable position, so in case of failure, the user would fall and cannot use the crutches to recover. Two safety approaches could be implemented. First, following special design rules and manufacturing processes, it is possible to ensure the system keeps operating despite a failure, which is also called fault-tolerant approach (IEC, 2011). An example of similar device using this approach is the Segway Personal Transporter (Segway Inc, United States), which has redundant sensors, control electronics, and motor windings (Segway Switzerlan, 2020). Another solution is to deploy an airbag to protect the user in case a fall is detected, as suggested by a ReWalk patent (Goffer and Tivon, 2014).

## Conclusion

A major result of this study was that postural adaptation strategies observed in healthy participants and elicited by standing in a passive exoskeleton could be ported onto an active exoskeleton with equivalent mobility. This conducted to the first full-mobilization exoskeleton able to balance during standing with only two degrees of freedom per leg. This could have important implications for the independence of individuals with paraplegia, their inclusion in social activities and their potential inclination to use an exoskeleton on a daily basis for the associated health benefits.

## Data Availability Statement

The original contributions presented in the study are included in the article, further inquiries can be directed to the corresponding author.

## Ethics Statement

Ethical review and approval was not required for the study on human participants in accordance with the local legislation and institutional requirements. The patients/participants provided their written informed consent to participate in this study.

## Author Contributions

JF conceived the idea and concept, designed the experiment, collected, analyzed and interpreted the data, and drafted the manuscript. RB and TV helped in conceiving the idea and concept. RB implemented the experiment, designed the controllers, participated in data acquisition, analysis and interpretation, and took part to the redaction. TV predominantly developed the two exoskeletons used in this study. AI and MB helped in drafting the manuscript and critically revising it. All authors contributed to the article and approved the submitted version.

## Conflict of Interest

The authors declare that this study received funding from Sonceboz SA. The funder was not involved in the study design, collection, analysis, interpretation of data, the writing of this article or the decision to submit it for publication.
